# Economic burden of vulvar and vaginal intraepithelial neoplasia: retrospective cost study at a German dysplasia centre

**DOI:** 10.1186/1471-2334-11-73

**Published:** 2011-03-22

**Authors:** Monika Hampl, Eduard Huppertz, Olaf Schulz-Holstege, Patrick Kok, Sarah Schmitter

**Affiliations:** 1 Universitätsfrauenklinik Düsseldorf, Moorenstr. 5, 40225 Düsseldorf, Germany; 2 Im Sand 4, 56412 Niedererbach, Germany; 3 Institut für Experimentelle Psychologie, Heinrich-Heine-Universtität, Universitätsstr. 1, 40225 Düsseldorf, Germany; 4 Sanofi Pasteur MSD GmbH, Paul-Ehrlich-Straße 1, 69181 Leimen, Germany

## Abstract

**Background:**

Human papillomavirus is responsible for a variety of diseases including grade 2 and 3 vulvar and vaginal intraepithelial neoplasia. The aim of this study was to assess parts of the burden of the last diseases including treatment costs. The direct medical resource use and cost of surgery associated with neoplasia and related diagnostic procedures (statutory health insurance perspective) were estimated, as were the indirect costs (productivity losses) associated with surgical treatment and related gynaecology visits for diagnostic purposes.

**Methods:**

Data from 1991-2008 were retrospectively collected from patient records of the outpatient unit of the Gynaecological Dysplasia Clinic, Heinrich Heine University, Dusseldorf, Germany. Two subgroups of patients were analysed descriptively: women undergoing one surgical procedure related to a diagnosis of vulvar and/or vaginal intraepithelial neoplasia, and women undergoing two or more surgical procedures. Target measures were per-capita medical resource consumption, direct medical cost and indirect cost.

**Results:**

Of the 94 women analysed, 52 underwent one surgical intervention and 42 two or more interventions (mean of 3.0 interventions during the total period of analysis). Patients undergoing one surgical intervention accrued €881 in direct costs and €682 in indirect costs; patients undergoing more than one intervention accrued €2,605 in direct costs and €2,432 in indirect costs.

**Conclusions:**

The economic burden on German statutory health insurance funds and society induced by surgical interventions and related diagnostic procedures for grade 2/3 vulvar and vaginal neoplasia should not be underrated. The cost burden is one part of the overall burden attributable to human papillomavirus infections.

## Background

Human papillomavirus (HPV) is responsible for a variety of diseases [1-3]. Discussion concerning HPV and associated epidemiology and disease burden, treatment and vaccination focuses predominantly on cervical cancer and cervical intraepithelial neoplasia (CIN) as a precursor of cervical cancer in women. While the incidence of cervical cancer has declined owing to the implementation of screening programmes, the incidence of vulvar intraepithelial neoplasia grades 2 and 3 (VIN 2/3) and vaginal intraepithelial neoplasia grades 2 and 3 (VaIN 2/3), primarily caused by HPV 16, have risen in recent decades. Data from the United States demonstrate a greater than fourfold increase in carcinoma *in situ *within a 30-year period [4]. In 2001, Anders *et al. *reported a VIN incidence of 7/100,000 women/year [5]. A German study established the presence of HPV in more than 90% of VIN 2/3 and VaIN 2/3 cases [6]. The increase in VIN and VaIN caseloads is attributable to a rising incidence of HPV infections and improved diagnostic procedures. The average age of affected women is between 40 and 50 years [4,7,8]. VIN is normally multifocal and present in combination with VaIN, CIN or anal intraepithelial neoplasia [9,10]. Severe VIN progresses to vulvar carcinoma in approximately 9% of untreated cases [11] and between 3.8% and 6.5% of treated cases [8,11]. Recurrence is common, being observed in approximately 30% of VIN lesions [12] and affected women suffer the individual distress caused by diagnostic and therapeutic procedures.

In Germany, medical care for women with VIN/VaIN is provided by primary care physicians, gynaecologists and dermatologists. For verification of diagnosis, further evaluation and treatment, patients are normally referred to an outpatient department of a hospital, generally a gynaecology department.

To assess the burden of these diseases, the costs for treatment of VIN/VaIN must be calculated because these are not currently available in Germany. The aim of the study was to estimate parts of the burden of disease on statutory health insurances (SHI) and society by:

1. Estimating the direct medical resource use and cost of surgery targeted to VIN 2/3 or VaIN 2/3 and related diagnostic procedures from a SHI perspective,

2. Approximating the indirect cost (productivity losses) generated by surgical treatment of VIN 2/3 and VaIN 2/3 and related visits to gynaecologists for diagnostic purposes.

## Methods

Data were retrospectively collected from patient records between 1991 and 2008 at one study site. The outpatient unit of the Dysplasia Clinic, Department of Obstetrics and Gynaecology at Heinrich Heine University, Dusseldorf, Germany, is specialised in the diagnosis and treatment of female lower genital tract diseases.

All patient entries included in the documentation of laser treatments were pre-screened by hand-searching to identify patients who had a (suspected) diagnosis of VIN 2/3 or VaIN 2/3 and had undergone surgical intervention. Patient records of eligible patients were checked in detail to ensure that they fulfilled the following criteria for the study:

• Had undergone at least one of the following surgical interventions, either as an outpatient or inpatient (reference intervention): laser excision, laser vaporisation, biopsy, inguinal lymphadenectomy, vulvectomy, vulvar resection;

• Had been subjected to a definite confirmed diagnosis of VIN 2/3 or VaIN 2/3 (as documented in the medical records of the study site);

• Lived in Germany at the time of intervention.

Patients with diseases other than grade 2 or 3 VIN or VaIN such as cancer or VIN 1, or those with incomplete documentation of interventions, were excluded from the study.

The reference intervention (i.e. the last or only surgical intervention within the observation period) had to be performed at the study site; prior interventions (inpatient or outpatient setting) could have taken place at the same site or at any other German hospital. Before and after surgical intervention, patients attended gynaecology outpatient departments of hospitals or office-based gynaecologists for diagnostic purposes (visit for diagnostic purposes). Diagnostic procedures were not standardised.

The following data were extracted from patient records using a case report form:

• Date of birth;

• Diagnoses;

• For inpatient surgery: date of admission; method of intervention; diagnostic procedures; length of stay in hospital [days], amount reimbursed by SHI [€];

• For outpatient surgery: date of surgical intervention; method of intervention; diagnostic procedures; amount reimbursed by SHI [€];

• For visits for diagnostic purposes: date; diagnostic procedures; amount reimbursed by SHI [€].

Two subgroups of patients were generated:

• **Subgroup I**: women who had undergone one surgery related to VIN 2/3 and/or VaIN 2/3 diagnosis (the most common patient category);

• **Subgroup II**: women subjected to two or more surgeries (representing more seriously affected patients).

**Figure 1:**
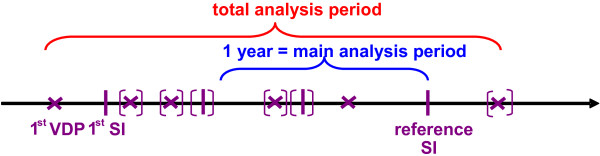
**Main analysis period and total analysis period for Subgroup II (Schematic diagram)**. Purple cross = visit(s) at dysplasia outpatient department or office based specialist for diagnostic purposes (VDP) Purple line = surgical intervention (SI) Purple brackets = optional VDP/SI In Subgroup I: main analysis period = total analysis period

Two time periods were analysed for each subgroup (Figure 1):

1. **Main analysis period **- a one year period before the reference intervention, reflecting the time horizon most relevant for VIN 2/3 or VaIN 2/3 surgery;

2. **Total analysis period **- the period from the first documented contact with a hospital/gynaecologist due to VIN 2/3 or VaIN 2/3 to the last reported contact, covering the full time horizon for which data were found in patient records at the study site. This period is sufficient to cover the total relevant cost.

Economic burden was estimated from two perspectives:

• The SHI perspective takes into consideration medical resource consumption and fees reimbursed by SHI;

• The social perspective refers additionally to productivity losses to society caused by treatment-related sick leave.

Target measures were per-capita medical resource consumption, direct medical per-capita costs and indirect per-capita costs. Medical resource consumption for the patients included in the study was measured by counting:

• The number of surgical interventions due to VIN 2/3 and/or VaIN 2/3 in an inpatient setting and an outpatient setting;

• The number of related outpatient visits for diagnostic purposes before and after surgical interventions.

Data relating to medical resource consumption were obtained from patient records.

Direct medical costs considered analogously were:

• Cost of inpatient surgery;

• Cost of outpatient surgery;

• Cost of visits for diagnostic purposes.

The direct medical costs were calculated using two approaches:

• The first approach was to use "historical" costs, fees reimbursed by SHI in the year of surgical intervention and visits for diagnostic purposes as recorded in patient records. This approach resulted in "historical" medical costs. For medical services not performed at the study dysplasia centre, or if no unit cost was available from patient records, the mean value of the respective cost category from the study site was taken as the unit cost.

To describe the trend followed by the three categories of direct medical costs over the time period examined, the respective mean values per case were normalised for six time periods: 1991-2002, 2003, 2004, 2005, 2006 and 2007-2008, using the z-score (z_ij _= (X_ij _- μ)/σ; i = 1, 2, 3; j = 1, 2,..., 6; X_ij_: mean of cost category i in time period j, μ: mean of all cost numbers; and σ: standard deviation of all cost numbers).

• To adjust for varying prices and fees over the time period, a scenario analysis was performed on the basis of unit cost in 2007 as found in patient records: median unit costs (base case), the lowest (best case) and the highest (worst case).

Productivity losses due to lost work days because of visits for diagnostic purposes and in association with surgical interventions were estimated as a proxy for indirect costs from society's perspective, based on the human capital approach. Per-capita costs were estimated as the mean duration (in days) per patient off work multiplied by a daily rate for "loss of productivity". Mean days off work per patient were conservatively estimated to be 0.5 days for surgery in an outpatient setting or attendance for diagnostic reasons, and the mean days on a ward for inpatient surgery (as found in the patient records). An average sick leave of five days after each surgical intervention was assumed. A daily rate of €90 (2006 value) was used, following the German Hanover Consensus, 3^rd ^edition [13].

The sample size was planned to be between 80 and 100 patients, as this number is known to be sufficient for precise estimations in cost studies.

Due to the study design and according to national legislative requirements a consultation at the ethics committee was not necessary. The national guideline for secondary data analysis [14] need to be complied as we did a retrospective chart review. Two exceptions from consultation by the ethics commission are mentioned in this guideline: 1) all data protection measures are fulfilled and 2) no relation to the primary data is planned. Exception 1 was fulfilled because according to the German data protection law, data from hospitalized cases may be used for scientific analyses by the hospital without special patient informed consent by the patients. Further to that, all patients in the study site signed a special form that their data may be used for scientific reasons. No relation to individual data was possible because all analyses of the primary data were performed at the study site and left the centre only in an aggregated manner. Therefore the second criterion was fulfilled, too.

SPSS version 13 G and MS Excel 2007 were used to perform the analyses. P-values presented here are based on the Welch test.

## Results

A total of 141 patients were eligible for the study; of these, 94 women fulfilled the inclusion criteria. Reasons for patient exclusion were no definite confirmation of VIN or VaIN diagnosis (20 cases), vulvar cancer (11 cases), no patient records (six cases), VIN 1 (four cases), condylomata (two cases), surgery not linked to VIN/VaIN diagnosis (two cases), melanoma (one case) and lichen sclerosis (one case).

The first documented surgical intervention was in January 1991 and the last was dated February 2008. Diagnostic visits were documented from March 1993 to February 2008.

Of the 94 women included in the analyses, 52 (55%) underwent one surgical intervention related to VIN 2/3 or VaIN 2/3 diagnosis and were assigned to subgroup I. 42 women (45%) underwent two or more interventions and were assigned to subgroup II.

### Demographics and basic characteristics

Patients in subgroup I had a mean total observation period of 0.53 years (range 0.01 - 3.64 years; median 0.13 years). The respective figures for subgroup II were 4.35 years (0.11 - 15.42 years; 3.38 years).

The baseline information is presented in table 1. VIN 3 was the dominant diagnosis in each subgroup. The range of patient ages had a normal distribution in each group and for both observation periods. Patients subjected to multiple surgical interventions (subgroup II) were younger on average than the patients who underwent one surgical intervention (subgroup I) (44 vs. 49 years; p = 0.026).

**Table 1:** Age, surgical interventions and visits to gynaecologists for diagnostic purposes

	Subgroup I			Subgroup II	
			
	Total analysis period	Main analysis period		Total analysis period	Main analysis period
**N**	**52**	**52**	**n**	**42**	**42**
Age at first SI (years) mean ± SD [range]	49.2 ± 13.8 [22-81]		Age at first SI (years) mean ± SD [range]	43.6 ± 10.3 [21-71]	46.8 ± 10.4 [21-77]
**Number of SI/pat**.	1		**Number of SI/pat**.	2 - 5	1 - 5
Mean SI/pat. ± SD	1		Mean SI/pat. ± SD	3.0 ± 1.19	1.74 ± 0.91
**Setting of SI**			**Setting of SI**		
Inpatient	30		inpatient	64	32
Outpatient	21		outpatient	42	31
n/a	1		n/a	20	10

Total	52		Total	126	73
Number of VDP mean ± SD [range]	2.33 ± 1.64 [1-7]	1.54 ± 0.54 [1-3]	Number of VDP mean ± SD [range]	6.26 ± 7.41 [1-41]	2.07 ± 1.24 [0-5]
**Diagnoses**			**Diagnoses in SI history**		

VIN2	7		VIN2	1	5
VIN3	40		VIN3	26	30
VIN2&VIN3	1		VIN2 - VIN3	11	3
VaIN2	2		VIN3 - VIN3&VaIN2	1	1
VIN3&VaIN3	2		VIN3 - VIN3&VaIN3	2	2
			VIN3&VaIN2	1	1
Total	52		Total	42	42

Subgroup I: women who underwent one VIN 2/3 and/or VaIN 2/3 related surgery.

Subgroup II: women subjected to two or more VIN 2/3 and/or VaIN 2/3 related surgeries.

Total analysis period: period from first VIN 2/3 or VaIN 2/3 related to the last reported contact.

Main analysis period: period of one year before the reference intervention.

SI: surgical intervention(s).

VDP: visit(s) at dysplasia outpatient department or office based specialist for diagnostic purposes.

In subgroup I each of the 52 surgical interventions was performed at the study site. For women who underwent surgery more than once (subgroup II), 15 interventions out of 73 (20.5%) in the main analysis period and 33 out of 126 (26.2%) in the total analysis period were performed at a site other than the study site.

Surgical interventions were predominantly carried out in an inpatient setting (87%) in the period between 1991 and 2002, but decreased (58%) in the period between 2003 and 2006. By 2007, most patients underwent surgical intervention as an outpatient (58%). Four surgical interventions, two for each setting, were carried out in 2008. The distribution of inpatient and outpatient settings was similar in the two subgroups across the total analysis period; patients in subgroup 1 were treated in an inpatient setting in 59% of cases and in an outpatient setting in the other 41%.

### Resource use

Laser vaporisation/excision after diagnostic biopsy for histology were the most common surgical interventions (78% in main analysis period). Superficial partial vulvar resections or superficial partial vulvectomies were the next most common (17%), often in combination with laser vaporisation (8%). Diagnostic procedures regularly accompanying an intervention were diagnostic biopsy, smear, sonography and ECG, colposcopy, haematology and chest X-ray. Patients who underwent more than one surgical intervention had a mean of 1.74 interventions during the main analysis period (inpatient setting 0.88; outpatient setting 0.86), and 3.0 interventions during the total analysis period (inpatient setting 1.81; outpatient setting 1.19; Table 2).

**Table 2:** Resource use and direct medical per-capita costs (incl. scenario analysis)

		Resource use: frequency per patient (patient records)	Direct medical per-capita costs		
			
			Scenario analysis varying unit costs of 2007		
		
		mean [units]	base case (median unit cost 2007)	best case (lowest unit cost 2007)	worst case (highest unit cost 2007)
**Total analysis period**					

**Total sample **	SI outpatient setting	**0.76**	€197.68	€90.54	€218.71
**n = 94**	SI inpatient setting	**1.13**	€1,082.99	€1,077.83	€2,182.83
	VDP	**4.09**	€366.42	€366.42	€487.24
	**Total**		€**1,647.09**	€**1,534.79**	€**2,888.78**

**Subgroup I **	SI outpatient setting	**0.41**	€106.64	€48.84	€117.99
**n = 52**	SI inpatient setting	**0.59**	€565.46	€562.76	€1,139.71
	VDP	**2.33**	€208.74	€208.74	€277.57
	**Total**		€**880.84**	€**820.35**	€**1,535.27**

**Subgroup II **	SI outpatient setting	**1.19**	€309.52	€141.76	€342.46
**n = 42**	SI inpatient setting	**1.81**	€1,734.70	€1,726.43	€3,496.40
	VDP	**6.26**	€560.83	€560.83	€745.75
	**Total**		€**2,605.05**	€**2,429.03**	€**4,584.61**

**Main analysis period**					

**Total sample **	SI outpatient setting	**0.61**	€158.66	€72.67	€175.55
**n = 94**	SI inpatient setting	**0.72**	€690.05	€686.76	€1,390.83
	VDP	**1.77**	€158.57	€158.57	€210.86
	**Total**		€**1,007.28**	€**918.00**	€**1,777.24**

**Subgroup I**	SI outpatient setting	**0.41**	€106.64	€48.84	€117.99
**n = 52**	SI inpatient setting	**0.59**	€565.46	€562.76	€1,139.71
	VDP	**1.54**	€137.97	€137.97	€183.46
	**Total**		€**810.07**	€**749.57**	€**1,441.16**

**Subgroup II **	SI outpatient setting	**0.86**	€223.69	€102.45	€247.49
**n = 42**	SI inpatient setting	**0.88**	€843.39	€839.37	€1,699.90
	VDP	**2.07**	€185.45	€185.45	€246.60
	**Total**		€**1,252.53**	€**1,127.27**	€**2,193.99**

Subgroup I: women who underwent one VIN 2/3 and/or VaIN 2/3 related surgery.

Subgroup II: women subjected to two or more VIN 2/3 and/or VaIN 2/3 related surgeries.

Total analysis period: period from first VIN 2/3 or VaIN 2/3 related to the last reported contact.

Main analysis period: period of one year before the reference intervention.

SI: surgical intervention(s).

VDP: patient visit(s) at dysplasia outpatient department or office based specialist for diagnostic purposes.

The mean number of outpatient visits for diagnostic purposes presented in Table 1 are based on a total of 80 visits for diagnostic purposes in subgroup I (main analysis period; 121 in the total analysis period) and 87 in subgroup II (main analysis period; 263 in the total analysis period).

The mean duration spent on a ward for inpatient surgery was 2.05 days for subgroup I and 4.9 days for subgroup II (main analysis period). The figures for the total analysis period were 2.05 and 4.58 days, respectively.

### Direct medical and indirect costs

Patient records presented individually documented "historical" costs (i.e. fees reimbursed by SHI in the respective year) per patient for surgical intervention and visits for diagnostic purposes. "Historical" costs per surgical intervention in the inpatient setting were variable over time, amounting to a mean of €1,344.39 per intervention (median €964.04; range: €484 to €13,764). Mean cost per intervention in an outpatient setting was €200.28 and the median was €239.62 (range €68.14 - €3,435.34). Mean cost per visit for diagnostic purposes was €54.31 and the median was €40.80 (range €40.67 to €119.13).

**Figure 2:**
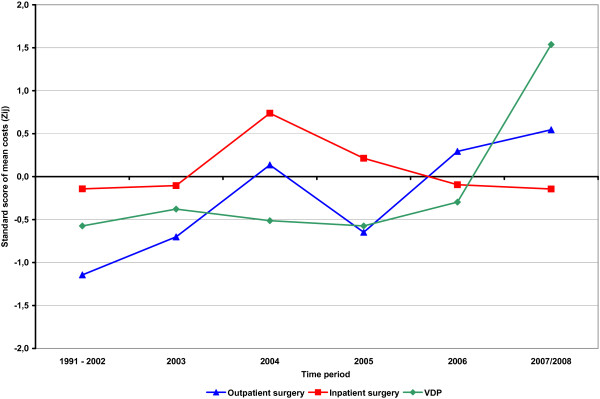
**Trend curve for mean direct medical cost per case**. Zij = (Xij - μ)/σ {i = 1, 2, 3; j = 1, 2, ..., 6} Xij: mean cost per case of category i in time period j VDP: visit(s) at dysplasia outpatient department or office based specialist for diagnostic purposes

Figure 2 demonstrates the trend curve for direct medical costs per case for each of the three categories over the 1991-2008 period. Cost per inpatient surgery remained relatively constant throughout the period. The cost of outpatient surgery rose during those years, and the cost of visits for diagnostic purposes increased from 2006 to 2007/2008.

Scenario analysis of direct medical cost used the average number of resource consumptions per patient (inpatient surgery, outpatient surgery and visits for diagnostic reason) as calculated from the numbers recorded in patient records for all three scenarios. These numbers were assigned the fees reimbursed by SHI to the study site in 2007. Median fees for "base case" calculations (lowest for "best case" and highest for "worst case") were:

• €958.40 per inpatient surgery (lowest €953.83; highest €1,931.71);

• €260.10 per outpatient surgery (lowest €119.13; highest €287.78);

• €89.59 per visit for diagnostic reason (lowest €89.59; highest €119.13).

Table 2 presents the resultant direct medical per-capita cost. The base case scenario resulted in overall direct costs of €1,007 (€1,253 in subgroup II and €810 in subgroup I) during the main analysis period. Per-capita direct cost was €1,647 (€2,605 and €881, respectively) for the whole period. Scenario analysis demonstrates that in a worst case scenario (i.e. using the highest documented cost for surgical intervention/visit for diagnostic purposes) the cost burden is approximately double that of the best case scenario (i.e. using the lowest documented costs). Costs for subgroup II were three times higher than subgroup I across the total analysis period and 1.5 times higher during the main period. Historical direct medical costs taken from patient records fit into the ranges given by the scenario analysis based on 2007 unit costs. Indirect costs (Table 3) ranged from approximately €650 in subgroup I during the main analysis period to approximately €2,450 in subgroup II for the total analysis period.

**Table 3:** Total per-capita costs

		Direct medical plus indirect per-capita costs			
		
		Direct medical costs	Productivity loss	Indirect costs	Total costs
		
		A	B	C = B * € **90**	D = A + C
		
		base case	mean [days]	Human capital approach	base case
**Total analysis period**					

**Total sample **	SI outpatient setting	€197.68	4.18	€376.13	€573.81
**n = 94**	SI inpatient setting	€1,082.99	9.98	€897.94	€1,980.93
	VDP	€366.42	2.04	€183.83	€550.25
	**Total**	€**1,647.09**	**16.20**	€**1,457.90**	€**3,104.99**

**Subgroup I **	SI outpatient setting	€106.64	2.26	€203.82	€310.46
**n = 52**	SI inpatient setting	€565.46	4.15	€373.24	€938.70
	VDP	€208.74	1.16	€104.71	€313.45
	**Total**	€**880.84**	**7.58**	€**681.77**	€**1,562.61**

**Subgroup II **	SI outpatient setting	€309.52	6.54	€588.40	€897.92
**n = 42**	SI inpatient setting	€1,734.70	17.35	€1,561.72	€3,296.42
	VDP	€560.83	3.13	€281.79	€842.62
	**Total**	€**2,605.05**	**27.02**	€**2,431.90**	€**5,036.95**

**Main analysis period**					

**Total sample **	SI outpatient setting	€158.66	3.34	€300.25	€458.91
**n = 94**	SI inpatient setting	€690.05	6.10	€548.71	€1,238.76
	VDP	€158.57	0.88	€79.47	€238.04
	**Total**	€**1,007.28**	**10.32**	€**928.43**	€**1,935.71**

**Subgroup I **	SI outpatient setting	€106.64	2.26	€203.82	€310.46
**n = 52**	SI inpatient setting	€565.46	4.15	€373.24	€938.70
	VDP	€137.97	0.77	€69.23	€207.20
	**Total**	€**810.07**	**7.18**	€**646.29**	€**1,456.36**

**Subgroup II **	SI outpatient setting	€223.69	4.70	€423.35	€647.04
**n = 42**	SI inpatient setting	€843.39	8.65	€778.67	€1,622.06
	VDP	€185.45	1.04	€93.21	€278.66
	**Total**	€**1,252.53**	**14.39**	€**1,295.23**	€**2,547.76**

Subgroup I: women subjected to one VIN 2/3 and/or VaIN 2/3 related surgery.

Subgroup II: women who underwent two or more VIN 2/3 and/or VaIN 2/3 related surgeries.

Total analysis period: period from first VIN 2/3 or VaIN 2/3 related to the last reported contact.

Main analysis period: period of one year before the reference intervention.

SI: surgical intervention(s).

VDP: patient visit(s) at dysplasia outpatient department or office based specialist for diagnostic purposes.

Estimated indirect costs were lower than direct costs in the base case of scenario analysis; with the exception of subgroup II in the total analysis period, where indirect costs marginally exceeded direct costs.

## Discussion

This study has presented initial data concerning the economic burden of VIN 2/3 and VaIN 2/3 surgery and related diagnostic procedures in Germany. The study site is one of the top dysplasia centres.

Cost burden associated with VIN 2/3 and VaIN 2/3 surgery and related diagnostic procedures is part of the overall burden due to HPV infections. Irrespective of whether VIN and VaIN are rare diseases, these results demonstrate that these costs should not be neglected in general consideration of costs for HPV-related diseases.

In the inpatient setting, "historical" costs per surgical intervention were variable over time. This variability is most likely to be due to changes in the German reimbursement system for inpatient care, with SHI reimbursement based on a per diem payment until 2004 and on DRGs thereafter. Another explanation could be the improvement in medical treatment options over time; we observed a shift from an inpatient to an outpatient setting, potentially resulting in the treatment of only severe cases in hospital.

Patients who underwent one surgical intervention generated approximately €1,560 in "total" costs, with patients undergoing more than one intervention (subgroup II) generating approximately €5,030. As expected, subgroup II had the highest per-capita resource consumption and costs because there were more surgical interventions, and the high unit cost of inpatient surgery added to direct costs. Available data indicated that subgroup II contained more seriously affected patients than subgroup I. Patients were younger at the date of first surgery than patients who underwent one intervention, and with respect to inpatient surgery, the average length of stay in hospital was twice as long as those in subgroup I. This indicates that women subjected to two or more surgical interventions require more complex treatment.

In 2008, Petry et al. published the results of a survey carried out in Germany that collected data concerning the cost of screening and treatment for cervical dyskaryosis. The study revealed total per-capita costs of €1,055 in the PAP III group (mean direct cost: €613; mean indirect cost: €442) and of €3,174 in PAP IV group (mean direct cost: €1,881; mean indirect cost: €1,293) [15]. In 2008, the same authors presented annual costs associated with genital warts in Germany, disclosing direct medical costs ranging from €315 (men, new cases) to €1,563 (women, resistant cases), and indirect costs due to sick leave ranging from €0 (men and women, new cases) to €232 (women, resistant cases) [16]. Cost burden studies in other countries including France and the United States [17-22] have used different approaches, making the results incomparable. For example, the average cost of CIN per episode of care was $1,709 (CIN 1 = $1,026; CIN 2 = $1,300; and CIN 3 = $3,235) in the United States [23].

There are limitations with this study as documents from a single site were used and the data cannot be seen as representative for Germany. Furthermore, owing to the low incidence of VIN 2/3 and VaIN 2/3 and the restriction to one study site, only 94 patients were included in the analysis. This is likely to have affected the validity of the results and should be kept in mind when interpreting and using these results for any further analyses. This is why we refrained from extrapolating them to the German population. However, the data give a basic idea of the dimensions of the burden in Germany where no cost data existed until now.

The costs presented are an excerpt of the total direct medical and indirect costs. Not all outpatient visits performed for diagnostic purposes are documented in patient records at the study site, and documentation of surgical interventions is not 100% complete. In addition, the study was unable to include the cost of screening for VIN and VaIN that did not lead to surgery. Further, the economic burden associated with vaginal and vulvar cancer was not included. Given that vulvar and vaginal cancers are rare diseases compared to intraepithelial neoplasia [5] and that we were limited to one study site, we expected during the planning of the study such analyses would not have led to valid results. Therefore, it was decided to not evaluate these costs at the beginning of the study. Hence, the costs presented here are likely to be underestimated, particularly for the total analysis period.

Indirect costs could only be estimated. Patient records did not contain information concerning sick leave days. The numbers used for calculation were based on expert opinion and should be viewed as data from experience. The human capital approach, a relatively simple method to quantify the monetary value of productivity losses with its pros and cons, has been discussed extensively in the literature [24-26]. A per diem productivity loss of €90, cited in the 3rd edition of Hanover Consensus13, is an approximation based on 2006. A precise 2007 figure would have been marginally higher.

## Conclusions

The economic burden on German statutory health insurance funds and society induced by surgical interventions and related diagnostic procedures for grade 2/3 vulvar and vaginal neoplasia should not be underestimated. The cost burden is part of the overall burden attributable to HPV infections. Discussion of HPV infections should not focus solely on cervical cancer and pre-cancers. Other HPV-induced tissue lesions and cancers should be taken into account.

## List of abbreviations

CIN: cervical intraepithelial neoplasia; DRG: Diagnosis Related Groups; HPV: human papillomavirus; PAP: Papanicolaou; SHI: statutory health insurances; SI: surgical intervention(s); US: United States; VaIN: vaginal intraepithelial neoplasia; VDP: visit(s) at dysplasia outpatient department or office based specialist for diagnostic purposes; VIN: vulvar intraepithelial neoplasia.

## Competing interests

MH declares that she has received an honorarium from Sanofi Pasteur MSD and GlaxoSmithKline for speaking at several scientific meetings and for acting as a scientific consultant. EH declares that he has received an honorarium from Sanofi Pasteur MSD for acting as a scientific consultant. SS is employed by Sanofi Pasteur MSD, who funded the study. OSH and PK declare that they have no competing interests.

## Authors' contributions

MH designed the study, provided advice on medical topics and participated in the writing of the manuscript. EH designed the study reviewed the results and wrote the manuscript. OSH participated in designing the study and collected the data. PK performed the statistical analysis. SS reviewed the results of the study, participated in the writing of the manuscript and was responsible for study coordination. All authors read and approved the final manuscript.

## Pre-publication history

The pre-publication history for this paper can be accessed here:

http://www.biomedcentral.com/1471-2334/11/73/prepub
